# Updating the Risk Stratification for Sudden Cardiac Death in Cardiomyopathies: The Evolving Role of Cardiac Magnetic Resonance Imaging. An Approach for the Electrophysiologist

**DOI:** 10.3390/diagnostics10080541

**Published:** 2020-07-31

**Authors:** Ourania Kariki, Christos-Konstantinos Antoniou, Sophie Mavrogeni, Konstantinos A. Gatzoulis

**Affiliations:** 1 Department of Cardiology, Onassis Cardiac Surgery Centre, 17674 Kallithea, Greece; sophie.mavrogeni@gmail.com; 2 First Department of Cardiology, National and Kapodistrian University of Athens, Hippokrateion General Hospital, 11527 Athens, Greece; CKAntoniou@hotmail.gr (C.-K.A.); kgatzoul@med.uoa.gr (K.A.G.)

**Keywords:** cardiomyopathy, sudden cardiac death, implantable cardiac defibrillators, ventricular tachycardia, ventricular fibrillation, cardiac magnetic resonance, electroanatomic mapping

## Abstract

The prevention of sudden cardiac death (SCD) in cardiomyopathies (CM) remains a challenge. The current guidelines still favor the implantation of devices for the primary prevention of SCD only in patients with severely reduced left ventricular ejection fraction (LVEF) and heart failure (HF) symptoms. The implantation of an implantable cardioverter-defibrillator (ICD) is a protective barrier against arrhythmic events in CMs, but the benefit does not outweigh the cost in low risk patients. The identification of high risk patients is the key to an individualized prevention strategy. Cardiac magnetic resonance (CMR) provides reliable and reproducible information about biventricular function and tissue characterization. Furthermore, late gadolinium enhancement (LGE) quantification and pattern of distribution, as well as abnormal T1 mapping and extracellular volume (ECV), representing indices of diffuse fibrosis, can enhance our ability to detect high risk patients. CMR can also complement electro-anatomical mapping (EAM), a technique already applied in the risk evaluation and in the ventricular arrhythmias ablation therapy of CM patients, providing a more accurate assessment of fibrosis and arrhythmic corridors. As a result, CMR provides a new insight into the pathological substrate of CM. CMR may help identify high risk CM patients and, combined with EAM, can provide an integrated evaluation of scar and arrhythmic corridors in the ablative therapy of ventricular arrhythmias.

## 1. Introduction

Cardiac plasticity permits the heart to adjust its function and structure in response to environmental stimuli such as the increase in the wall thickness or the dilatation of the ventricles due to pressure or volume overload conditions [1]. The term cardiomyopathy (CM) is used to describe an inhomogeneous group of disorders affecting the function and the structure of the myocardial wall without an obvious triggering factor such as arterial hypertension, coronary artery disease, valvular disease or congenital heart disease. The European Society of Cardiology (ESC) in 2008 classified CMs in five groups according to their functional and morphological characteristics (hypertrophic cardiomyopathy (HCM), dilated cardiomyopathy (DCM), arrhythmogenic right ventricular cardiomyopathy (ARVC), restricted cardiomyopathy (RCM) and an unclassified group) [2]. The prevalence of CMs differs significantly among these groups, with HCM and DCM being the most common (1:250/500) and ARVC (1:2000/5000) and RCM (unknown prevalence) being the rarest [3]. It is worth noting that although the term of ischemic cardiomyopathy (ICM) is used to describe patients with myocardial dysfunction caused by severe coronary disease, it is, by definition, not considered to be a ”real” CM according to the ESC since myocardial hypoperfusion is the cause of the dysfunction. 

Patients diagnosed with any kind of CM are threatened by a variety of complications including progressive heart failure, need for cardiac transplantation or sudden cardiac death (SCD). Even if manifestations such as an episode of heart failure decompensation give time to seek medical help and are manageable, SCD leaves no room for reaction. SCD can be divided into two major categories—deaths that occur as a consequence of a mechanical complication, for instance, myocardial rupture or left ventricular outflow tract obstruction in HCM and deaths that have a malignant arrhythmic origin [4]. The latter cases are more likely to have an autopsy negative SCD since the post-mortem analysis of the cardiac conduction system is not routinely feasible given that it is technically demanding [5]. As an epidemiological hint, in the United States, up to 300,000 cardiac deaths occur suddenly every year. The majority of them have an ischemic etiology (75%) followed by CMs (15%) [6]. More precisely, in DCM, studies suggest that 30% of deaths occur unexpectedly. Among them, patients with preserved functional status (NYHA class I and II) are more prone to SCD (50–60% of their overall mortality) than patients in a worse clinical status who mainly die due to heart failure complications [7]. Undoubtedly, the primary prevention of SCD demands that medical teams are a step forward in patients’ therapeutic management. 

Accurate and straightforward arrhythmic risk stratification algorithms for the primary prevention of major arrhythmic events (MAEs)—including, in this article, SCD and both sustained ventricular tachycardia (VT) and fibrillation (Vf)—constitute the holy grail of clinical electrophysiology. An effective and efficient approach allows for the rational targeted allocation of life-saving implantable cardioverter defibrillators (ICDs), with the expected benefit outweighing any device related complication and adverse events (e.g., infections and inappropriate shocks) [8]. Based on results from seminal trials, most of which were published approximately 15 years ago [9,10,11,12,13,14,15,16,17], left ventricular function, assessed in terms of ejection fraction (LVEF), and functional status (NYHA classification), dominate American [18] and European [19] primary prevention guidelines regarding both ischemic (ICM) and nonischemic dilated cardiomyopathy (NIDCM).

The aforementioned studies were not designed to stratify patients according to arrhythmic risk but rather, based on their LVEF; consequently, they only demonstrated that moderately-to-severely impaired LVEFs were associated with high arrhythmic risk, without including patients with mildly-to-moderately impaired contractility. Not unexpectedly, a two-fold disparity between ICD allocation and MAE occurrence in the primary prevention population has been noted, with an ever increasing trend, given that the majority of sudden deaths in the community occurs in people with mildly impaired LVEF [20,21], and in modern ICD registries [22], the rates of appropriate ICD activation are low (2.6% at 30 months), even at low LVEFs.

As contemporary studies [20,21,22] raise skepticism about the true role of LVEF in the prediction of malignant arrhythmias, the introduction of new parameters in the daily practice for the risk stratification of patients is a necessity for the near future. Cardiac magnetic resonance (CMR) is a highly effective imaging modality for the characterization of myocardial tissue as well as for the anatomical and the functional evaluation of the heart [23]. Several CMR findings are associated with arrhythmogenic substrates in CMs, with the late gadolinium enhancement (LGE) quantification and distribution pattern being the most well established [24,25,26,27,28,29,30,31,32,33,34]. The addition of CMR in the field of risk stratification for SCD of the CM patients is promising.

The aim of this review is to present the CMR imaging patterns of the arrhythmogenic substrate, studied up to date in CMs, and examine the role of CMR findings in the clinical decision making of the electrophysiologist. 

## 2. CMR Basics

The diagnostic imaging of the human body, independently of the modality used, enables the creation of contrast between tissues. The signal source that serves as contrast for magnetic resonance imaging (MRI) is the response of hydrogen atoms to the magnetic field. Each hydrogen atom is composed of a proton which carries its own spin. The human body does not spontaneously create magnetic signal since spins are oriented randomly [35]. By placing the human body in a strong uniform magnetic field, the spins are forced to align in a parallel or anti-parallel way along the field, creating a new equilibrium with slightly more atoms choosing the parallel orientation. The next step is the interruption of this provoked equilibrium by a radiofrequency pulse (RF) applied in the magnetic field for a specific amount of time. Hydrogen atoms absorb and reemit the radiofrequency received in a different manner that depends on their surroundings. After the appliance of the pulse, as the spins return to the previous equilibrium, the phenomenon of ”relaxation” begins. T1 relaxation, or spin-lattice relaxation time, is the recovery of the longitudinal net magnetization vector, and it occurs due to the exchange of energy between the spins and the lattice. T2 relaxation time or spin–spin relaxation is the recovery of the transverse net magnetization vector, and it occurs due to spin–spin interactions and their progressive dephasing [36].

The heart, as a constantly beating organ, which is also affected by respiration movements, makes CMR measurements challenging because of multiple artifacts. Techniques such as the Look Locker (LL) sequence, the Modified Look Locker Inversion Recovery Sequence (MOLLI), the shortened MOLLI (shMOLLI) and the Saturation recovery single-shot acquisition sequence (SASHA) overcome these difficulties by using protocols of supplement excitation RF pulses to extract T1 measurements and have significantly improved the reproducibility of the method [37].

Regarding T1 mapping, it is created by the accumulation of multiple T1 measurements from many heart beats, all at the same phase of the cardiac cycle. Each T1 value is represented by a pixel in the map. T1 mapping images the entire myocardium and does not separate extracellular from cellular environments. The deposition of lipids, water, proteins or iron change T1 measurements and reveal early histological changes [38,39]. Native T1 is a CMR parameter that does not require the use of contrast and, subsequently, is suitable for patients with renal impairment. 

When CMR imaging is being supplemented with intravenous contrast agents, the information derived is multiplied. Gadolinium-based contrast agents (GBCAs) are the most widely used agents approved for use [40].They are well-tolerated with extremely low allergic reactions and side effects (0.01%) and have a minimum impact on renal function. Nephrogenic Systemic Sclerosis (NSF), is a rare side effect of GBCAs and has only been reported in patients with renal impairment [41]. Although deposits of gadolinium have been identified in the brains of patients without renal disease who received multiple doses, the clinical relevance of this finding remains to be clarified [42,43]. GBCAs cannot insert the intracellular compartment due to their high molecular weight. Consequently, their distribution is possible only to the extracellular space-intravascular compartment and interstitial space. 

**Figure 1:**
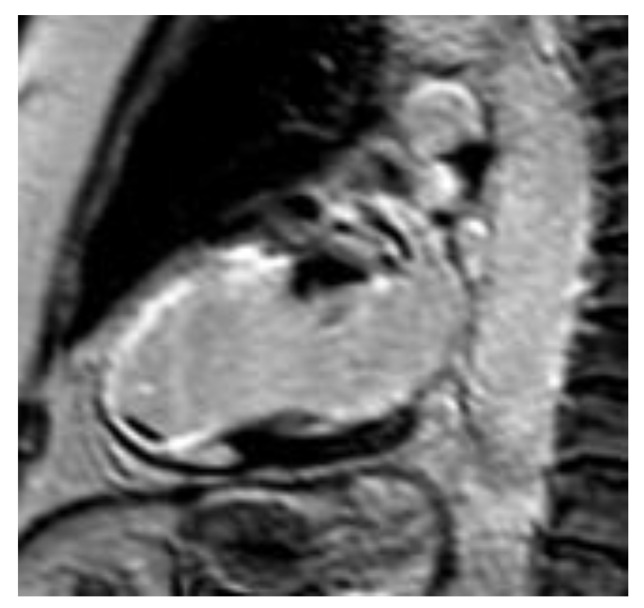
‘’Bright is dead’’. Inversion recovery image showing extensive LGE area due to anterior myocardial infarction. During late imaging, a gadolinium-based contrast agent (GBCA) is present in areas of fibrosis, as mentioned in the text. Τhe bright area in the anterior and apical wall represents scar tissue.

The paramagnetic properties of gadolinium lead to a shorten T1 relaxation time in these areas. The most valuable information extracted by the use of GBCA is its pattern of removal from tissues. Normally, after its first distribution, a GBCA is progressively washed out. In areas of myocardial fibrosis, the wash out is delayed since the extracellular space is expanded, the capillary density is reduced and the distance between myocardial cells is augmented. Imaging the heart ”late” after the gadolinium has been washed out from healthy tissue can identify these areas with pathological response indicative of replacement fibrosis. It is noticeable that, by definition, late gadolinium enhancement (LGE) (Figure 1) expresses the difference between two areas and is not useful in diffuse myocardial disease, where the entire myocardium is affected and no healthy areas exist to serve as a control [44].

**Figure 2:**
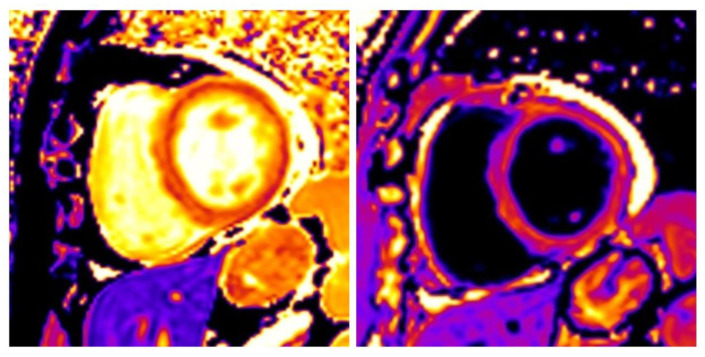
Native and postcontrast T1 mapping in the midventricular short axis level of the left ventricle (LV) in a patient with inflammatory cardiomyopathy. The left image was derived before the administration of a GBCA. The myocardial wall is shown in an inhomogeneous deep orange color which corresponds to pathologically elevated native T1 values. The right one was taken after the administration of the agent. It is worth noting that postcontrast T1 values are influenced by factors other than tissue characteristics such as the agent’s dosage and the time of the imaging. An algorithm that combines native and postcontrast T1 values allows us to calculate a more tissue specific parameter, the ECV, in areas of interest.

Last but not least, extracellular volume (ECV) is another useful parameter reflecting histological changes early in the CMs’ course, independently of their cause. It is measured by the native and postcontrast T1 mapping also including the hematocrit level [45,46] (Figure 2).

The quantification of ECV by CMR is an excellent noninvasive method as the measurements are highly correlated with the results of biopsies [47,48]. ECV is useful when the myocardium is being infiltrated by diffuse diseases such as amyloidosis or diffuse fibrosis that cannot be evaluated with LGE distribution, demanding areas of healthy tissue for comparison and also thicker areas of collagen deposition in order to be visible [38,49].

A common issue affecting all contrast-based scar detection lies in the definition of scar and its subtypes, given that it hinges on relative signal intensity. There is not a universal cutoff over which the high signal intensity in LGE-CMR reflects scar tissue. Subsequently, the definition of scar varies between different studies and researchers. Two major approaches have been pursued, one defining scars based on increased signal intensity (versus a healthy remote area) and the other defining healthy tissue based on less pronounced signal intensity (versus the maximal intensity observed at a dense scar region). A few of the most popular scar definitions in studies are the followings: areas with signal intensity >6 standard deviations (SD) higher than the average intensity of remote areas [50], or with signal intensity ≥50% of the maximal within infarct zone value [51,52,53], or signal intensity >2SDs above the value for normal myocardium [54]. At the same time, border scars (grey zone) can be defined as having an intensity of either 35% to 50% [53] or <60% [55] of the above value.

## 3. CMR Imaging Patterns Reveal the Arrhythmogenic Burden in Cardiomyopathies

### 3.1. Nonischemic Dilated Cardiomyopathy

The most well established parameter of an arrhythmogenic substrate in NIDCM is the LGE imaging [24,25,26,27,28,29,30,31]. The existence, the localization but not the quantification of LGE are all strong independent predictors for ventricular arrhythmias, SCD and hospitalization in all ranges of LVEF [56,57,58]. Precisely, septal LGE carries the worst prognosis even if the fibrotic area is restricted. Moreover, the coexistence of septal with free wall LGE as well as a subepicardial pattern of LGE are all additive risk factors for fatal arrhythmias [56,57,58,59]. Interestingly, concerning the inducibility of ventricular arrhythmias during programmed ventricular stimulation (PVS), the absence of LGE is an excellent predictor of noninducibility of monomorphic but not polymorphic VT/VF [29,50,60].

Furthermore, T1 mapping and ECV are also valuable tools in the arrhythmic risk stratification of NIDCM [46,61,62,63,64]. ECV and postcontrast T1 mapping correlate with the severity of systolic dysfunction [61]. An increase in ECV is detected early, even before the systolic impairment, reflecting the pathological substrate of the myocardium. Except from the early reveal of the disease, ECV measurements serve also as an independent prognostic factor for cardiovascular death, heart failure hospitalization and appropriate ICD firing [46,47]. However, in patients who cannot receive intravenous contrast agents, ECV measurements can be successfully replaced by native T1 mapping which is a strong independent predictor of an adverse outcome such as all cause mortality, heart failure death and hospitalization in NIDCM [62,64].

Finally, CMR derived left ventricular longitudinal strain is a newly proposed marker of prognosis in patients with NIDCM with conflicting results [65,66]. In one study, the prognostic value of longitudinal strain was even superior to other markers such as LVEF, NT-proBNP and LGE mass [65]. However, other studies failed to confirm this finding [67].

### 3.2. Ischemic Cardiomyopathy

The scar tissue following an acute coronary syndrome (ACS) constitutes the most common arrhythmogenic substrate in ICM [68,69,70]. Although malignant arrhythmias occurring early after an ACS are caused by abnormal automaticity and triggered activity of hypoperfused myocardial cells, after the formation of the scar, mechanisms of reentry dominate [71].

Focal myocardial fibrosis detected by LGE imaging substantially increases the risk for life threatening arrhythmias in ICM [32,33,34,72]. Histologically, a myocardial scar is not always a uniform lesion. In the dense core of the scar, areas of fibrosis are interrupted by viable fibers that serve as slow conduction pathways. In addition, the tissue surrounding the core of the scar is a ‘grey zone’ of hypoperfused myocardium with arrhythmogenic properties [73,74,75]. The heterogeneity of the scar as well as the size of the border zone assessed by CMR parameters are all predictors of malignant arrhythmias and appropriate ICD firings in ICM [76,77,78].

Regarding the role of T1 mapping and ECV measurements in the arrhythmic risk stratification of ICM, there are a few studies, including both ischemic and nonischemic CM patients, suggesting a correlation between their pathological values and an increased arrhythmic burden [79,80].

Here, it should be highlighted that another highly effective risk stratification approach with absolute negative predictive value, and a positive predictive one of 22% over a mean follow up of 32 months, is that of PVS response in post-myocardial infarction (MI) patients with a LVEF ≥ 40% having at least one out of seven noninvasive risk factors (NIRFs) present on ambulatory and signal averaged electrocardiography [81]. This approach did not incorporate any abnormal CMR data of the corresponding post-MI population although a probable association can coexist in these patients [82]. Such a two step combined noninvasive/invasive risk stratification approach holds promise for a significant, yet until recently unrecognized post MI patient population at risk, to be addressed through cost effective and readily available algorithm [83].

### 3.3. Hypertrophic Cardiomyopathy

In a rare divergence between American and European guidelines [84,85], primary prevention in hypertrophic cardiomyopathy (HCM) is based either on the presence of 1 out of 3 major clinical risk factors (SCD history, marked left ventricular (LV) hypertrophy, unexplained syncope) with the presence of LGE upon CMR and outflow tract obstruction acting as risk modifiers in cases of nonsustained VT and abnormal blood pressure response to exercise (AHA) or on the calculated 5-year risk of MAEs (ESC–ICD strongly indicated when ≥6%). Notably, there is considerable debate on the value of the latter approach, with contradictory evidence published [86,87]. 

Indeed, there has been considerable debate concerning the value of the latest European risk score [84,88] regarding arrhythmic events in HCM, as exhibiting high specificity but low sensitivity [89]. This is a population where protection from arrhythmic death has been claimed to lead to an almost normal life expectancy [90], although recent surveys [91] cast doubt on this, reporting an increased mortality even among such well protected patient cohorts. The reverse is true for the currently undergoing major reshaping [92] American criteria, that have excellent sensitivity yet rather low specificity [89], with patients experiencing more inappropriate shocks and complications than receiving appropriate therapies [93,94]. Thus, CMR has long been thought as a potential tie-breaker in the clinical decision-making process [95,96].

The presence of scar, imaged by LGE in HCM patients is considered to be a strong independent predictor for ventricular arrhythmias, ventricular remodeling, all-cause mortality and cardiac death [72,97,98,99,100]. The extent of myocardial LGE involvement has been proposed as a better risk stratifier, with cutoffs oscillating from as low as 10% [101], to as high as 20%, with the mean value of 15% attaining wider acceptance [96]. The pattern of LGE distribution, patchy with multiple foci or diffuse, does not carry an additional risk [100]. However, diffuse myocardial fibrosis identified by pathological postcontrast T1 mapping is correlated with episodes of NSVT on Holter monitoring of HCM patients [102].

A relatively common finding during CMR imaging of HCM patients is the appearance of areas of T2-high signal spotted within LGE. Although its clinical significance is not well established, a study of 81 HCM patients concluded that high T2 signal is another parameter for VT manifestation in this group [103]. Concerning the role of ECV, a study incorporating the measured global ECV in the already suggested HCM risk-SCD score of the European society of cardiology (ESC) was associated with a significant improvement in the diagnostic accuracy of the score offering a better risk stratification in this disease [104].

### 3.4. Inflammatory Cardiomyopathy

Inflammatory CM is the result of an inflammatory process due either to infective or autoimmune causes. Myocardial oedema and fibrosis as a result of macro-, micro-vascular coronary artery disease, vasculitis and autoimmune myocarditis may lead to SCD in patients with autoimmune diseases, even if the left ventricular ejection fraction (LVEF) is preserved [105]. These changes in the myocardial substrate of patients with autoimmune rheumatic diseases (ARDs), imaged in CMR as elevated median T1 mapping values, pathological T2 mapping and increased ECV values, were all predictors for malignant arrhythmias in a study of 61 patients with ARDs and preserved LVEF [105].

Between various ARDs, polymyalgia rheumatica, the commonest rheumaticdisease in elderly people, may present with SCD, as a result of chronic inflammation. Several studies demonstrated that besides promoting structural heart disease, inflammatory activation may also be per se arrhythmogenic, via cytokine-mediated effects on cardiac electrophysiologic properties. Furthermore, there is increasing evidence that inflammation is a risk factor for QTc prolongation and associated life-threatening arrhythmias, specifically torsade de pointes (TdP) [106]. Additionally, rheumatoid arthritis (RA) patients have twice the risk of SCD, compared with non-RA patients, due to an increased incidence of VT/VF. Although the pathophysiologic background of the proarrhythmic substrate in RA is rather complicated, it seems that chronic systemic inflammation can introduce arrhythmias either by promoting the development of ICM or by altering the electrophysiological properties of the heart. Therefore, the tight control of the inflammatory process is probably the most effective intervention to decrease the arrhythmic risk in RA patients.

Autonomic dysfunction, potentially leading to SCD, even in the absence of relevant symptoms or reduced LVEF, was documented in systemic lupus erythematosus, idiopathic juvenile arthritis patients and children with type 1 diabetes mellitus [107,108,109]. Sporadic VT/VF has also been observed in dermato-, poly-, and inclusion body myositis patients [110]. Finally, SCD was observed in various infiltrative diseases such as sarcoidosis and systemic sclerosis [111]. Specifically, in systemic sclerosis, the presence of oedema and LGE are predictive factors for arrhythmia development [112].

## 4. The Complementary Role of CMR and Electroanatomical Mapping in Ventricular Tachycardia Ablation

A significant limitation of VT ablation is its variable success rate. The HELP-VT study evaluated VT ablation outcomes among patients with ICM and NIDCM, demonstrating similar success rates in the short term, but with significant reappearance of the arrhythmia in the long term in the latter [113]. The absolute VT noninducibility has been claimed as a prerequisite for long term success [113]. The differences in the arrhythmia substrate are a major determinant of this failure in NIDCM, since the isthmus and critical parts of the circuit are located in regions difficult to map and ablate such as the epicardium [114].

**Figure 3:**
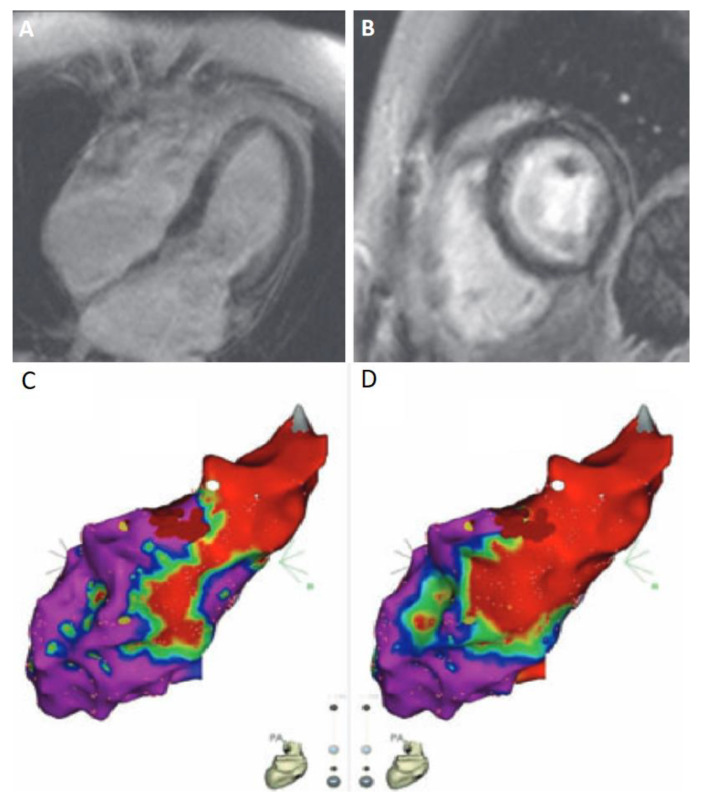
Joint application of cardiac magnetic resonance (CMR) and electro-anatomical mapping (EAM) in the case of a 43 year old female diagnosed with Behçet’s disease and high burden of symptomatic ventricular extrasystoles (≈20,000 on 24 h ambulatory electrocardiography), without LV dysfunction. (**A**,**B**) T1-weighted sequence CMR with obvious LGE both at the left ventricular lateral wall (midmyocardial) and subendocardially (diffuse pattern, not corresponding to coronary artery territory). Four chamber (**A**) and short axis (**B**) views. (**C**,**D**) Electroanatomical map of the LV using the CARTO3^®^ system (Biosense-Webster, Diamond Bar, CA, USA) depicting a low voltage area (non-purple color) in the same segments, using both bipolar (**C**) and unipolar (**D**) settings. Successful ablation of the arrhythmia was achieved following radiofrequency energy application at the basal lateral wall, an area with abnormal unipolar but not bipolar voltage values, suggesting a nonsubendocardial arrhythmogenic focus [116].

Electroanatomic mapping (EAM) is the currently used approach to identify the arrhythmiologic substrate. However, in EAM the arrhythmogenic substrate is identified indirectly by collecting local voltage amplitudes of the myocardium. This method has some drawbacks: (a) it is time consuming, (b) it lacks sensitivity for scar substrates deep in the mapped surface and (c) it lacks specificity for scar detection in the case of poor catheter contact or thin myocardium. Therefore, the need for new strategies to define arrhythmogenic scar substrates is relevant [115]. CMR examination before or during EAM could reveal these difficult areas and potentially enhance the ablation’s success rate. An example of the complementary approach to the incorporation of CMR in interventional electrophysiology is depicted in Figure 3.

The first necessary step in this algorithm is to incorporate CMR into 3D-EAM in order to transfer anatomical data from one software environment to the other. Potential causes of a mismatch include different rhythms during different image acquisition timings, as well as different loading conditions, leading to slight geometric alterations in chamber geometry [117], as it has been shown in several studies [55,118,119]. Regarding the association between scar features and measured voltages, a correlation between both bipolar and unipolar values with scar nature (dense/border, transmurality) has been reported [118]. However, it is worth noting that a bipolar voltage threshold of <1.5mV (usual threshold for border-zone tissue) did miss dense scars with <75% transmurality and nontransmural border scars, potentially explaining why some VT isthmuses were not detected by substrate 3D-EAM and were not ablated [118].

In one of the first studies, to evaluate the integration of CMR to achieve the demanding task of scar definition in a clinical setting [120], this imaging technique facilitated conducting channel characterization and demarcation of possible entrance sites, allowing for a more targeted and comprehensive isolation. The ability of CMR to accurately detect subendocardial conducting channels was also confirmed in another study [55], where 81% of them could be identified by CMR prior to 3D-EAM. Similar results have been reported [54] with an impressive 92.2% of 3D-EAM points lying within a 5mm distance of the imported CMR-formed shell. Additionally, all critical sites for premature ventricular contractions (PVCs) and VT isthmuses were detected within the LGE-defined scar.

In a more radical approach, the field of magnetic resonance electrophysiology (MR-EP) [119], alternatively known as interventional CMR (iCMR), has been developed with the CMR scanner now forming an integral, rather than a complementary/auxiliary, part of the 3D-EAM. The approach is still theoretic, with proof of concept animal studies having been carried out to validate the functionality of prototype integrated MR-EP systems [121,122] with no currently existing human studies [117]. More precisely, a scanner can be used, virtually in lieu of a fluoroscopy machine [123] to:

- Generate anatomic images during the procedure. Consequently, any registration mismatch errors are avoided. The operator uses the CMR scanner virtually in the same manner as a fluoroscope, with an attached pedalfor image acquisition whenever e.g., tissue characterization is needed [117].
- Locate indwelling ablation catheters [121,122,123,124].
- Produce substrate maps to be compared with those derived from the “classical” EAM module and improve area of interest targeting.
- Evaluate the formation of permanent, irreversible lesions [124,125,126] prior to catheter withdrawal. Regarding real-time lesion formation assessment, acute LGE grossly overestimates the effect (ECV expansion), while thenoncontrast method, using fast T1- and T2-weighted imaging [127,128,129,130] has only been able to acutely detect a minority (~30%) of lesions. CMR thermography is a newly introduced parameter with promising results [42,131,132].

## 5. Future Perspectives in the Arrhythmic Risk Stratification in Cardiomyopathies

A multidisciplinary approach for the risk stratification of ICM and NIDCM patients seems to be more suitable than the proposed oversimplified one based on LVEF so far, in order to better define their complex substrate and support individualization. Towards this direction, several efforts have been made to update primary prevention arrhythmic risk stratification in ICM and NIDCM with rather well maintained LVEF, either by assessing the presence of an arrhythmogenic substrate through a two-step approach with noninvasive risk factor presence leading to PVSin both diseases [81,89,133] or by the use of imaging [56,59,134,135,136] in the latter. Such preliminary combinational approaches are associated with encouraging results [81,133,137] for the detection of a high risk NIDCM cohort with low LVEF and 3year event rates similar to that of a secondary prevention population based on the ESTIMATED score, a score derived from the combination of abnormal CMR findings with various clinical and electrocardiographic NIRFs. 

In this direction, two prospective observational studies are currently underway: The CMR-Guide trial [138] (NCT01918215) will define the role of CMR in the primary prevention of SCD in ICM and NIDCM patients with well maintained LVEF>35%. After assessing the presence of either an ischemic or nonischemic LGE pattern, patients will be randomized to receive either an implantable loop recorder or an ICD. The ReCONSIDER study [139] (NCT04246450) will incorporate CMR findings, namely LGE presence along with a variety of other NIRFs, leading to invasive PVS in NIDCM patients across the LVEF spectrum in a two step approach.

The question of which is the best risk stratification approach among HCM patients with a low European risk score and/or a single conventional noninvasive risk factor present, remains a highly debatable issue. On the contrary, there is solid evidence that, in the presence of multiple NIRFs and/or a high European risk score, the justification of ICD insertion is well supported [89]. Although electrophysiology testing (ET) has received a class III recommendation for the risk stratification of HCM patients [19], it has an excellent predictive ability for HCM patients with a single NIRF, such as the presence of nonsustained VT or unexplained syncope, by revealing either a potentially malignant ventricular arrhythmogenic substrate and/or an underlying conduction system disease [89]. Whether a combinational multifactorial, CMR and ET-inclusive, approach is appropriately suited for this rather large subgroup of HCM patients is currently unknown [140].

## 6. Conclusions

There is an urgent need to update our risk stratification criteria for the appropriate and timely selection of the ICD candidates among CM patients at all LVEF spectrum values. In this direction, CMR imaging may play a significant role. Indeed, LGE quantification and pattern of distribution, as well as abnormal T1 mapping and ECV, can further enhance our ability to identify patients susceptible to life threatening arrhythmogenesis. Currently, more sophisticated approaches combining a variety of noninvasive and invasive modalities are underway in the new era of the arrhythmic risk stratification among CM patients even at early heart failure stages with less impaired LV function. Furthermore, CMR in combination with EAM may assist in the effectiveness of demanding interventional EP procedures not uncommonly applied among such seriously affected high risk patients.
